# Metabolic costs of capital energy storage in a small‐bodied ectotherm

**DOI:** 10.1002/ece3.2861

**Published:** 2017-03-12

**Authors:** Blaine D. Griffen

**Affiliations:** ^1^ Department of Biological Sciences and the School of the Earth, Ocean, and EnvironmentUniversity of South CarolinaColumbiaSCUSA

**Keywords:** capital breeder, *Carcinus maenas*, energy storage, metabolic rate, poikilotherm

## Abstract

Reproduction is energetically financed using strategies that fall along a continuum from animals that rely on stored energy acquired prior to reproduction (i.e., capital breeders) to those that rely on energy acquired during reproduction (i.e., income breeders). Energy storage incurs a metabolic cost. However, previous studies suggest that this cost may be minimal for small‐bodied ectotherms. Here I test this assumption. I use a laboratory feeding experiment with the European green crab *Carcinus maenas* to establish individuals with different amounts of energy storage. I then demonstrate that differences in energy storage account for 26% of the variation in basal metabolic costs. The magnitudes of these costs for any individual crab vary through time depending on the amount of energy it has stored, as well as on temperature‐dependent metabolism. I use previously established relationships between temperature‐ and mass‐dependent metabolic rates, combined with a feasible annual pattern of energy storage in the Gulf of Maine and annual sea surface temperature patterns in this region, to estimate potential annual metabolic costs expected for mature female green crabs. Results indicate that energy storage should incur an ~8% increase in metabolic costs for female crabs, relative to a hypothetical crab that did not store any energy. Translated into feeding, for a medium‐sized mature female (45 mm carapace width), this requires the consumption of an additional ~156 mussels annually to support the metabolic cost of energy storage. These results indicate, contrary to previous assumptions, that the cost of energy storage for small‐bodied ectotherms may represent a considerable portion of their basic operating energy budget. An inability to meet these additional costs of energy storage may help explain the recent decline of green crabs in the Gulf of Maine where reduced prey availability and increased consumer competition have combined to hamper green crab foraging success in recent years.

## 1 Introduction

Energy storage is a ubiquitous and integral aspect of biological systems with important ecological consequences. Energy storage has long been known to occur in a diverse range of organisms, including humans (e.g., Ogden et al., 2006) and other mammals (e.g., Beck, Bowen, & Iverson, 2003), birds (e.g., Blem, 1976), reptiles (e.g., Derickson, 1976), amphibians (e.g., Fitzpatrick, 1976), marine invertebrates (e.g., Lawrence, 1976), insects (e.g., Arrese et al., 2001), plants (e.g., Murphy, 1993), and algae (e.g., Khozin‐Boldberg & Cohen, 2011). Energy storage is not only broadly distributed across the tree of life, but also plays an important role in a similarly broad range of ecological processes. For instance, energy storage can have carry‐over effects that influence individual success through sequential life stages or across disparate spatial and temporal stages (Harrison, Blount, Inger, Norris, & Bearhop, 2011). Energy storage plays a critical role in hibernation (Dark, 2005), torpor (e.g., Walsh, Foster, & Moon, 1983), or other periods when resource intake is reduced (e.g., Cherel, Robin, & Le Maho, 1988). And energy storage plays an important role in important life‐history processes, such as metamorphosis (e.g., Manzon, Youson, & Homes, 2015) and reproduction (e.g., Mendo, Semmens, Lyle, Tracey, & Molschaniwskyj, 2016), with organisms often differing in the timing of energy storage relative to these energy‐demanding events (i.e., capital vs. income breeding strategies, Stephens, Boyd, McNamara, & Houston, 2009).

While energy storage is ubiquitous in its occurrence and pervasive in its influence, it also carries with it costs (Jöhnsson, 1997). At the physiological level, energy storage requires the maintenance of supportive tissues as well as the storage compounds themselves (Pond, 1981). Consequently, metabolic rate often increases with body mass, and may even increase more than proportionally with body mass, suggesting storage costs that are quite high (Daan, Masman, Strijkstra, & Verhulst, 1989; Piersma, Cadée, & Daan, 1995). At the ecological level, energy storage can decrease the speed and/or increase the costs of swimming (e.g., Billerbeck, Lankford, & Conover, 2001), flying (reviewed in Witter & Cuthill, 1993), and running (e.g., Trombulak, 1989). This decreased locomotor capability can subsequently hamper the ability of individual organisms to escape predators (e.g., Kullberg, Fransson, & Jakobsson, 1996; MacLeod, Barnett, Clark, & Cresswell, 2006; MacLeod et al., 2007), thus imposing a fitness cost. As a result, individual organisms appear to behave so as to moderate or control the amount of stored body fat below maximal levels (squirrels: Blake, 1972; dolphins: MacLeod et al., 2007; reviewed for birds in Witter & Cuthill, 1993), and it has recently been suggested that nonhuman animals may even exercise to keep fit (Halsey, 2016).

However, while the costs of energy storage appear to apply broadly, these costs are not expected to be equivalent for all groups of organisms. For instance, the costs of energy storage are expected to increase with the duration that these stores are carried, and should therefore be higher for capital breeders that store up reproductive energy in advance of the reproductive season relative to income breeders that finance reproduction with energy acquired during the reproductive season (Jöhnsson, 1997). This reasoning has been used to posit that capital breeders may be at a disadvantage in low‐productivity environments or in highly competitive environments where energy intake is insufficient to support the extra metabolic cost of maintaining energy stores (Stephens et al., 2009). However, even within capital breeders as a group, the cost of energy storage is expected to vary with body size (Stephens et al., 2009) and should differ between endotherms and ectotherms (Bonnet, 1998). Overall, the costs of energy storage are expected to be relatively low in small‐bodied ectotherms.

Here I examine the assumption that energy storage costs are low for small‐bodied ectotherms by examining the costs of energy storage in the European green crab *Carcinus maenas* (referred to hereafter as the green crab). This species is a relatively small‐bodied (<15 g dry mass for adult females) endothermic poikilotherm and is primarily a capital breeder (Griffen, Altman, Hurley, & Mosblack, 2011), but also increases reproductive output with increased food consumption during the reproductive period (Griffen, 2014), suggesting that it employs a mixed capital‐income strategy. This species is a globally distributed invasive species (Carlton & Cohen, 2003), and its reproductive strategy varies between locations (Yamada, 2001), likely due to differences in temperature patterns in different inhabited regions. Given the geographical variation in the number and timing of egg clutches produced by this species each year (Yamada, 2001), it is possible that the relative use of capital vs. income strategies could potentially vary for this species geographically. However, within its invaded region in the western Atlantic where animals for this study were collected, this species remains inactive in shallow subtidal habitats during the winter season and then extrudes eggs during early spring (Berrill, 1982) before active foraging has begun, thus necessitating energy storage over winter and the use of a primarily capital breeding strategy at this site.

Energy storage in the green crab, and in crabs in general, includes a combination of short‐ and long‐term strategies. Short‐term energy stores in crabs generally take the form of glucose within the hemolymph (Oliveira, Rossi, Kucharski, & Da Silva, 2004) and glycogen within muscle tissue (Briffa & Elwood, 2004) or the hepatopancreas (Parvathey, 1971). In contrast, energy is stored long term via lipid deposits within the hepatopancreas (Oliveira et al., 2004; Vonk, 1960), a midgut digestive organ that, in addition to lipid storage, produces digestive enzymes and functions in the absorption and digestion of food. Previous work has shown that green crab long‐term energy storage commonly ranges between 2% and 13% of body mass (Griffen et al., 2011).

Here I examine three questions. First, how does long‐term energy storage scale with variation in dietary intake? Second, what is the cost of long‐term energy storage in terms of changes in basal metabolic rate? And third, how do these costs integrate over longer timescales that incorporate environmental fluctuations in temperature and known changes in energy storage that reflect the seasonal timing of reproduction?

## 2 Methods

### 2.1 Laboratory induction of energy storage

I examined the magnitude of energy storage costs for the green crab using data from a previously published experiment designed to examine the role of diet in reproductive performance of green crabs (see Griffen, 2014 for a full description of experimental details—here I report only those aspects of the study related to energy storage and the costs of that storage). Forty female crabs (33.6–48.9 mm carapace width) were collected from the New Hampshire coast in early May of 2009. At this time of year in the Gulf of Maine, most reproducing green crabs are still carrying eggs (Berrill, 1982). In an attempt to select crabs with the lowest energy stores possible, I selected only nongravid crabs with a green carapace, indicating that they had recently molted (Styrishave, Rewitz, & Anderson, 2004). The experiment varied the total amount of food offered to individual crabs (four food levels: 0.2, 0.4, 0.8, 1.6 g every other day) and the proportion of that food that was animal tissue (tilapia) or algae (the red alga *Chondrus crispus*) (five levels: 0.0:1.0, 0.25:0.75, 0.5:0.5, 0.75:0.25, 1.0:0.0). Individual diet treatments were maintained for 8 weeks and the precise amount of animal and algal tissue consumed was measured throughout that time. The treatments described above were crossed orthogonally, yielding 20 different diet combinations that were each presented to two crabs. However, these two crabs should not be viewed as experimental replicates because each crab differed in its daily food choices irrespective of the food option provided (i.e., no two crabs had the exact same average daily consumption of animal tissue or algae over the course of the experiment). Thus, each of the 40 crabs in the experiment had a unique diet and resulting unique energy storage over the 8‐week experiment. The experiment included algae in the diet in order to determine its impact on reproductive performance (described in Griffen, 2014); however, algal consumption had no impact on energy storage and so, for simplification, the analyses presented here focus only on animal consumption.

During the sixth week of the experiment, I measured the metabolic rates of each crab. Crabs were starved for 24 hr prior to measuring metabolic rates to ensure that differences between individuals reflected differences in resting metabolic rates and not specific dynamic action associated with different experimental diets (Robertson, Meagor, & Taylor, 2002). During these measurements, crabs were held in air‐tight containers filled with sea water at 14°C. Oxygen content was measured every 10 min for 1 hr using a YSI 52CE dissolved oxygen probe. I used the slope from a regression of oxygen concentration vs. time to determine the metabolic rate of individual crabs (mg O_2_ g^−1^ dry weight hr^−1^).

At the conclusion of the experiment, I dissected each crab and removed the hepatopancreas. I dried the hepatopancreas and the rest of the body separately for 72 hr at 70°C. I then used the dry mass of the hepatopancreas as a proportion of the total dry mass of a crab (i.e., the hepatosomatic index, HSI) as a size‐independent metric of energy storage. I then used a linear model to examine how the HSI varied as a function of the average daily mass‐specific amount of animal tissue consumed (gram of tissue per gram of crab). One crab died during the experiment, and several crabs at the time of metabolic rate measurements had initiated a molt. These crabs were not used in the analysis of metabolic rates, leaving just 32 crabs for this analysis.

Next, I further explored the relationship between body size, energy storage, and crab mass. The mass of a crab may change with body size (larger crabs will weigh more) and with the amount of energy stored as lipid in the hepatopancreas. I therefore determined the relative contribution of each of these factors to the mass of the experimental crabs using a linear model (body mass relationships were linear within the relatively small size range of crabs used in this experiment) with mass as the response variable, and with carapace width and hepatopancreas mass as the predictor variables. To further tease apart the effects of body size (carapace width) and energy storage, I conducted a partial regression analysis. I used the residuals from regressing hepatopancreas mass against carapace width as the response variable, and the residuals from regressing body mass on carapace width as the predictor variable. This allowed me to examine how the mass of experimental crabs varied with mass of the hepatopancreas, after accounting for differences due to body size (carapace width) alone.

I next determined the metabolic cost of energy storage by examining the increase in metabolic rate as a result of energy storage. I used AIC to compare three linear models, each with metabolic rate (mg O_2_ hr^−1^) as the response variable. One of the models included hepatopancreas mass and crab body mass (minus the hepatopancreas) as predictor variables. The other two models, respectively, included only hepatopancreas mass and only body mass (minus the hepatopancreas) as the sole predictor variable.

### 2.2 Annual metabolic requirement calculations

I calculated the daily metabolic requirements of a representative adult female crab (assumed CW* *=* *45 mm). Reproductive female green crabs range from 31 to 67 mm CW (personal observations), with a mean of 46.5 mm CW in the Gulf of Maine (Berrill, 1982). I then summed these daily estimates over 1 year to determine the annual metabolic requirements. I performed this calculation twice, once for a crab that stored energy in its hepatopancreas following the assumed pattern described below, and once assuming no energy storage at all. I then used the difference between these two calculated annual metabolic requirements as an estimate of the metabolic cost of energy storage. There are countless possible scenarios for metabolic costs of individual crabs that will differ with crab size and the dynamics of energy storage, which are determined by daily foraging success, daily amount of time spent active, relative use of intertidal and subtidal habitats, etc. The scenario provided here is simply one plausible scenario that is based on known foraging and behavior patterns of green crabs.

Metabolic requirements for poikilotherms are determined largely by environmental temperature and body mass. I included the impacts of both factors as follows. I approximated the daily mean water temperature (*T*) using the following equation and assumed that this temperature reflected body temperature:

(1) $$T=a+\frac{b\cos2\pi \times \left(d-e\right)}{c}$$

where *a* (value 11.5) and *b* (value 8.5) were chosen by repeated trial and error to shift the curve upward and to stretch it vertically (Matthiopoulos, 2011) in order to mimic the appropriate range of seasonal sea surface temperatures at Hampton Beach, NH, over the last 30 years (from surf‐forecast.com), *c* (value 365) stretched the curve horizontally to produce one complete temperature cycle per year, *d* is the Julian day of the year for which the temperature is being calculated (i.e., 1–365), and *e* (value 240) shifted the curve horizontally so that the warmest SST occurred on day 240 (i.e., August 28).

For a given size crab, body mass is strongly influenced by the mass of energy stored in the hepatopancreas. The mass of the hepatopancreas, in turn, varies with food consumption (Griffen, Vogel, Goulding, & Hartman, 2015), resulting in seasonal fluctuations in body mass that result from a combination of seasonally variable consumption rates and metabolic processes that use this stored energy (e.g., growth and molting, reproduction) (Kennish, 1997). Previous work has shown that depletion of energy stores via reproduction in early spring (April on the New Hampshire coast) in green crabs results in an HSI of ~0.02 (Griffen et al., 2011). I therefore used April 30 as the start date for daily calculations and assumed an HSI of 0.02. I used data from the experiment described above on the laboratory induction of energy storage to derive the following equation to predict body mass (*M*) as a function of crab carapace width (CW) and hepatopancreas mass (*H*) (*R* ^2^ **=* *.87):

(2) M=0.225×CW+5.216×H−5.799

Using this equation and an assumed CW of 45 mm, I calculated the mass of a crab with no hepatopancreas (*M*_0_) as 4.35 g. I then assumed an initial HSI of 0.02 (i.e., *H *=* *0.087 g) and calculated the initial body mass for a crab on April 30.

I calculated the daily change in body mass via energy storage or depletion using a piecewise function, as follows. Green crabs only feed normally at temperatures as low as 6–7°C, below which feeding stops (Cohen, Carlton, & Fountain, 1995; Eriksson & Edlund, 1977). Additionally, green crabs commonly consume ~3% of their own body weight in food per day (Griffen, 2014), and on a diet of mussels, this results in a growth rate of 0.2% body mass per day (Mente, 2003). I therefore assumed that food consumption resulted in a linear increase in energy storage at a rate of 0.2% per day (i.e., HSI increased at 0.002 per day) until a maximum HSI of 0.12 was reached, which is consistent with the highest HSI observed in green crabs, generally just before the start of vitellogenesis (Griffen et al., 2011). Once this maximum HSI was reached, I assumed that additional foraging simply maintained this level of energy storage until the temperature dropped below 5°C, at which point crabs were assumed to stop feeding for the winter. During the nonfeeding winter period, I assumed that energy storage decreased at a rate of 0.02% per day, given reduced metabolic rates at cold temperatures (Newll, Ahsnullah, & Pye, 1972). These calculations are therefore given by:

(3) $$H_{x+1}=\left\{\begin{array}{cc} H_{x}+0.002M_{0} & \text{for}\;T>5\;\;\text{and}\;\;\text{HSI}<0.12\\ H_{x} & \text{for}\;T>5\;\;\text{and}\;\;\text{HSI}=0.12\\ H_{x}-0.0002M_{0} & \text{for}\;T\leq 5 \end{array}\right.$$

where the subscript *x* indicates day. I assumed that before active foraging resumed in the spring the crab extrudes its eggs (Berrill, 1982), depleting the remainder of its stored energy in the process and reducing the energy content of the hepatopancreas to its starting point at HSI* *=* *0.02. Finally, I assume that minimal foraging during this cold time of year was just sufficient to maintain the existing energy storage at HSI* *=* *0.02 until the end of the 1‐year calculation on April 29. Each of these assumptions allowed me to calculate the anticipated body mass of the crab as it changed daily as a result of feeding and metabolic activities that altered the hepatopancreas mass throughout an entire year.

Next, I used the daily calculated body mass and temperature to calculate daily metabolic costs. I measured metabolic rates of crabs as part of the experiment described in the preceding section; however, those measurements were made at a single temperature, whereas the calculations here were performed over a range of temperatures reflecting the annual variation in temperature in the Gulf of Maine. McDonald, Holsman, Beauchamp, Dumbauld, and Armstrong (2006) derived an equation describing the mass‐specific resting metabolic rate (*R*) of green crabs as a function of temperature (*T*) from empirical data provided by Newll et al. (1972). I used this same function after first converting it from the original units of ml O_2_ g^−1^ AFDW hr^−1^ (where AFDW is ash‐free dry weight) to calories expended using a conversion rate of 3.25 cal mg^−1^ O_2_ consumed (Elliott & Davison, 1975) and then converting from calories to kJ g^−1^ AFDW day^−1^, yielding the following equation (*R* ^2^ **=* *.51):

(4) R=0.037×T+0.947

I then used *R* to calculate the daily metabolic energy expenditure (*E*_*x*_) as a function of the crab body mass on day *x*, which changed with hepatopancreas mass as described above, and the daily temperature:

(5) $$E_{x}=R_{x}\times M_{x}$$

I further modified the projected energy expenditure because active metabolic rates of green crabs are ~3× higher than resting metabolic rates (Wallace, 1972). I therefore determined total daily metabolic expenditure (*Y*_*x*_) as:

(6) $$Y_{x}=P\times 3E_{x}+\left(1-P\right)\times E_{x}$$

where *P* is the proportion of time spent active (assumed to be 0.25). Finally, I also modified the daily metabolic expenditure during the winter nonfeeding time period to give the seasonal‐dependent daily metabolic expenditure (*Z*_*x*_) because green crab metabolic rates decline by 40% after 7 days of not eating, and by an additional 20% after 21 days of not eating (Marsden, Newell, & Ahsanullah, 1973):

(7) $$Z_{x}=\left\{\begin{array}{cc} Y_{x} & \text{for}\;\;T>5\;\;\text{within the last}\;7\;\text{days}\\ 0.6\times Y_{x} & \text{for}\;\;T\leq 5\;\;\text{for the last}\;7\;\text{days}\\ 0.4\times Y_{x} & \text{for}\;\;T\leq 5\;\;\text{for the last}\;21\;\text{days} \end{array}\right.$$

I then summed the seasonal‐dependent daily metabolic expenditure over each of the calculated days to determine the total metabolic expenditure for the entire year (*E*_annual_) of a crab that incurs the metabolic cost of energy storage:

(8) $$E_{\mathrm{annual}}=\sum_{x=1}^{365}Z_{x}$$

For comparison, I also calculated *E*_annual_ for a crab that does not store energy in its hepatopancreas, and therefore, HSI remained constant at 0.02 throughout the year. All other aspects of the calculation were identical to those described above. I used the difference between the metabolic costs of this constant weight crab and those of the crab whose body mass varies normally as described above to determine the increased metabolic cost (kJ) required to support energy storage. As indicated above, the size range of reproductive crabs in the Gulf of Maine spans 31–67 mm CW. I therefore repeated the above calculations with a 31‐mm crab and a 67‐mm crab to examine whether the observed trends were size‐dependent.

### 2.3 Energy content of hepatopancreas and eggs in field‐captured individuals

Finally, I also determined the energy content of the hepatopancreas and of a single egg in order to provide some context for the efficiency of storing energy. I examined the energy content of the hepatopancreas and eggs taken from individual green crabs sampled from Odiorne Point State Park, New Hampshire, on 27 and 29 April 2009, during the height of the reproductive season (*n *=* *86 for hepatopancreas and *n *=* *81 for eggs). Sizes of subsamples for hepatopancreas and eggs ranged from 0.008 to 0.195 g, and their energy content (joules/g of sample) was determined by combusting each sample in a Parr 6725 semi‐micro oxygen bomb calorimeter. For the hepatopancreas, I scaled the subsample up to determine the energy content of the entire hepatopancreas, and then examined how the energy content of the hepatopancreas (response variable) varies with hepatopancreas mass (predictor variable) using a linear model. For eggs, I fit a linear model with sample energy content as the response variable and sample mass as the predictor variable. I did not include an intercept in this model because a sample with a mass equal to zero must have zero energy content. I then used the slope from this model, multiplied by the mass of a single egg (13.73 μg, Griffen, 2014), to determine the energy content of a single egg.

## 3 Results

### 3.1 Laboratory induction of energy storage

**Figure 1:**
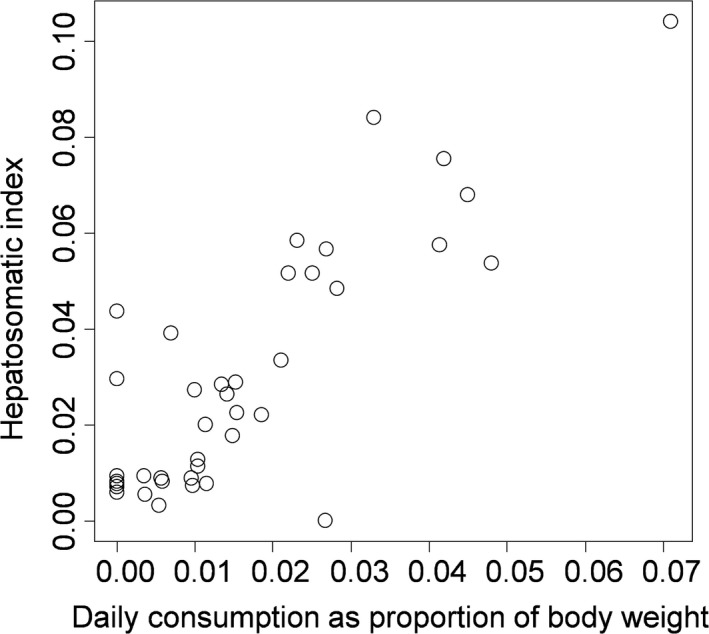
Energy storage in the hepatopancreas (hepatosomatic index, HSI) of *Carcinus maenas* as a function of the mean percent of their own body mass in animal tissue consumed daily during an 8‐week laboratory feeding experiment

**Figure 2:**
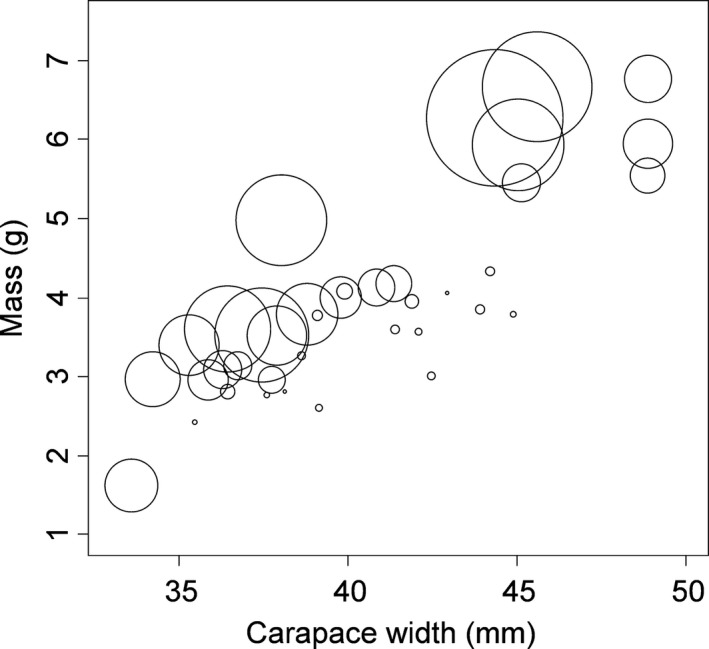
Mass of individual *Carcinus maenas* at the conclusion of an 8‐week feeding experiment as a function of the carapace width (*x*‐axis) and the weight of the hepatopancreas (relative weight shown by circle size)

**Figure 3:**
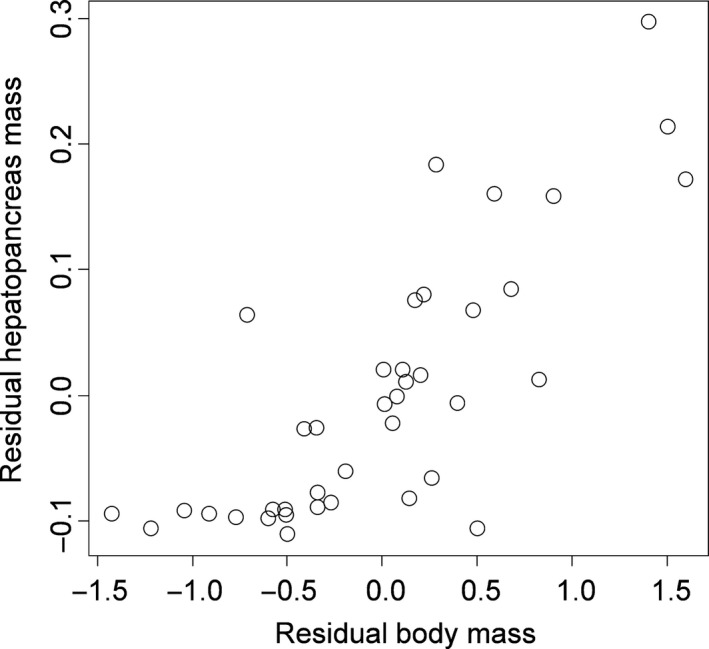
Relationship between hepatopancreas mass and body mass of experimental crabs after accounting for the effects of crab body size (carapace width)

Experimental diet had a strong impact on energy storage as approximated using the HSI. Specifically, energy storage increased strongly with the mass‐specific consumption of animal tissue (linear model parameter estimate 1.32 ± 0.15, *t *=* *8.91, *p *≪ .0001, adj. *R* ^2^ **=* *.67, Figure 1). Crab body mass increased with both the carapace width of the crab (linear model parameter estimate 0.23 ± 0.02, *t *=* *13.01, *p *≪ .0001, Figure 2) and with the mass of the hepatopancreas (linear model parameter estimate 5.22 ± 0.69, *t *=* *7.61, *p *≪ .0001, multiple adjusted *R* ^2^ **=* *.87, Figure 2). Partial linear regression indicated that residual body mass increased with residual hepatopancreas mass after controlling for differences in body size based on carapace width (linear model parameter estimate* *=* *5.22 ± 0.68, *t *=* *7.71, *p *≪ .0001, adj. *R* ^2^ **=* *.61, Figure 3).

**Figure 4:**
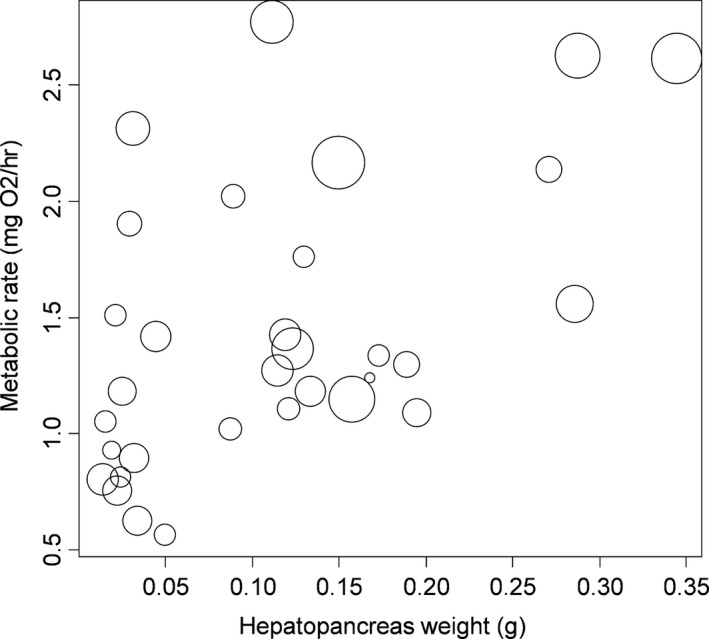
Resting metabolic rate of *Carcinus maenas* as a function of the amount of energy stored in the hepatopancreas (*x*‐axis) and the nonhepatopancreas body mass (relative circle size)

Resting metabolic rate was best explained by the model that included both hepatopancreas mass and nonhepatopancreas body mass (AIC* *=* *49.23), rather than the model with just hepatopancreas mass (AIC* *=* *52.64) or the model with just nonhepatopancreas body mass (AIC* *=* *52.49). Based on the model with both predictor variables, metabolic rate increased strongly with hepatopancreas mass (linear model parameter estimate 2.40, *t *=* *2.28, *p *=* *.030, Figure 4) and increased weakly with nonhepatopancreas body mass (linear model parameter estimate 0.18, *t *=* *2.31, *p *=* *.028, Figure 4). However, there was still a considerable amount of variation in resting metabolic rate that was not explained by either of these variables (multiple adj. *R* ^2^ **=* *.35).

### 3.2 Annual metabolic requirement calculations

**Figure 5:**
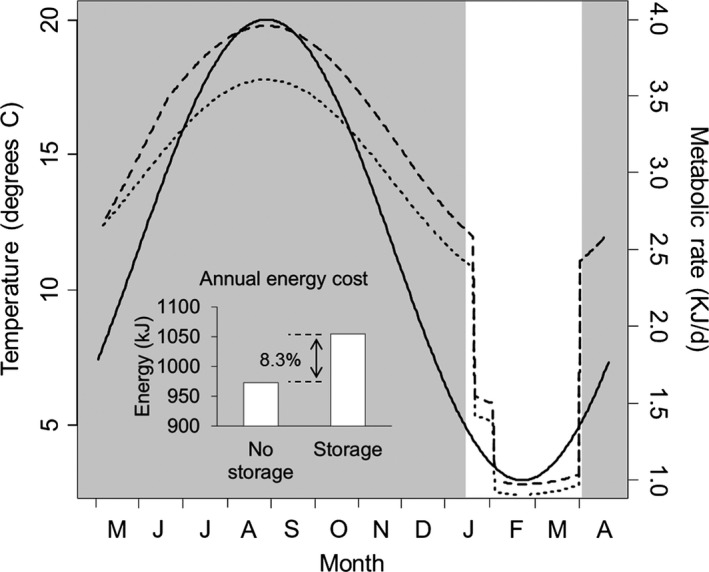
Model predicted metabolic costs of *Carcinus maenas* that stores energy in the hepatopancreas (dashed line) compared to control crab that does not store energy (dotted line) over an annual cycle as a function of temperature (solid line) and body mass including energy storage. Shaded region of graph shows portions of the year where temperature >5°C and so crabs will actively feed. Inset shows sum of daily metabolic rates over a single year for crab that stores energy vs. the control crab that does not store energy

The temperatures calculated here indicate that there are 283 days throughout the year when the temperature exceeds the minimum required for active feeding (i.e., >5°C). The calculated daily energetic expenditure varied with daily temperature (Figure 5), but also varied with crab mass, and thus, the amount of energy a crab was storing. Overall, the calculated annual energetic costs for a 45‐mm CW crab that follows the pattern outlined here of energy storage and expenditure throughout the year was 1,055 kJ, compared to just 973 kJ for a hypothetical crab that does not store any energy and therefore has a constant biomass (~8.3% difference, Figure 5 inset). The difference between these, or 82 kJ, is the estimated annual cost of energy storage for a crab this size. These calculated energetic costs do not include extra energy demands associated with molting, which generally occurs annually in adult green crabs. Repeating these calculations with a 31‐ and a 67‐mm CW crab demonstrated that the overall difference in annual metabolic costs between energy storing crabs and hypothetical control crabs that do not store energy increases with crab size, but the percentage change (8.3% metabolic cost increase for storing energy) is independent of crab size.

### 3.3 Energy content of hepatopancreas and eggs in field‐captured individuals

**Figure 6:**
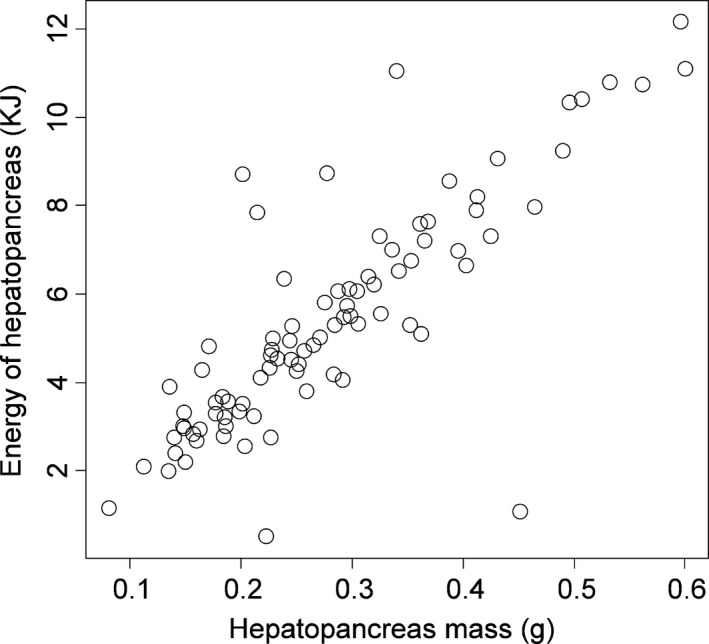
Energy content of the hepatopancreas from field‐captured *Carcinus maenas* as a function of hepatopancreas mass

The mass of the hepatopancreas in crabs collected from the field during the reproductive season varied more than sevenfold (range: 8.11–60.02 mg). Overall, the energy stored in the hepatopancreas increased linearly with the mass of the hepatopancreas (linear model parameter estimate 18.25 ± 1.36, *t *=* *13.41, *R* ^2^ **=* *.68, Figure 6). For eggs, the energy content of the sample increased with sample size with a slope of 22.239 kJ/g (*t *=* *22.25, *p *≪ .0001, *R* ^2^ **=* *.87). Multiplying this by the mass of a single egg yields an energy content of 0.305 joules per egg.

## 4 Discussion

I have shown that energy storage increases in the green crab *C. maenas* with increasing animal tissue consumption and that maintaining this stored energy incurs a metabolic cost. I have further shown that this metabolic cost of energy storage represents an overall increase in basal metabolic costs of ~8.3%, relative to a hypothetical crab that did not store energy, over an annual basis. It should be recognized that the calculations here are only approximations, given the large number of assumptions used, the resulting error propagation, and the fact that these calculations represent just one possible scenario for the time course of energy storage over a year. Nevertheless, the calculations above are based on the documented ecology and physiology of this species and on reasonable assumptions and therefore should provide a reasonable approximation of the metabolic costs of energy storage. Fat storage is also known to increase basal metabolic rates in other organisms. For instance, 6.7% of between‐individual variation in basal metabolic rates in humans is due to fat storage (Johnstone, Murison, Duncan, Rance, & Speakman, 2005).

As described in the Introduction, energy storage may incur metabolic and ecological costs via multiple mechanisms. I have demonstrated a metabolic cost via increased resting metabolic rate with energy storage; however, the underlying mechanism responsible for this increased metabolic cost was not examined and remains unknown. Further, in this study, I have not examined potential ecological costs, such as hampered movement or decreased predator avoidance. Nor have I examined potential benefits of energy storage for growth, reproduction, or physiological performance. The results here therefore only examine one aspect of the consequences of energy storage in a small‐bodied ectotherm and should not be viewed as the complete, or net, costs incurred by individuals as a consequence of energy storage.

The annual energetic costs for a crab that stores energy as calculated above (1,055 kJ, 82 kJ of which were expressly in support of energy storage) can be translated into required mussel consumption. McKinney, Glatt, and Williams (2004) give the energy content of mussels (*Mytilus edulis*) as 19.71 kJ/g, and the allometric function to determine the dry mass of mussels from length, where mass* *=* *0.00001 × length^3.42^. The model calculation used here assumed a crab with a 45 mm CW. This size of crab most efficiently consumes small mussels, <20 mm shell length (Elner & Hughes, 1978). Therefore, assuming consumption of 15‐mm‐long mussels, and using the dry weight:wet weight ratio of 0.25 (Ricciardi & Bourget, 1998), yields a wet mass of 0.026 g, and an energy content of 0.519 kJ per mussel. Therefore, a 45‐mm CW crab must consume nearly 2,033 mussels (of 15 mm length) annually, of which approximately 156 expressly support energy storage costs. Again, there is considerable error propagation in these calculations; however, they provide a general estimate of consumption requirements.

This level of consumption may be unsustainable within a Gulf of Maine environment that is changing very rapidly. The green crab invaded this region approximately 200 years ago (Say, 1817), but has declined in rocky intertidal areas over the last two decades following the introduction of a second invasive crab, the Asian shore crab *Hemigrapsus sanguineus* (Griffen et al., 2011; Kraemer, Sellberg, Gordon, & Main, 2007; Lohrer & Whitlatch, 2002). Rapid declines in mussels, and in other animal prey, are common within intertidal habitats following the arrival of the Asian shore crab (Kraemer et al., 2007; Lohrer & Whitlatch, 2002). Green crabs may also suffer from a loss of food abundance that is independent of the impacts of the Asian shore crab. The abundances of mussels and other sessile animals have steadily declined throughout the Gulf of Maine, dropping by >60% over the last four decades, apparently due to warming conditions (Sorte et al., 2017). Indeed, surface waters in the Gulf of Maine are warming faster than 99% of the global ocean, increasing by 0.03°C/year over the last 35 years, and accelerating to 0.23°C/year for the last decade (Pershing et al., 2015).

Not only does warming apparently decrease food availability, it also increases metabolic costs for poikilotherms. If the rate of temperature increases over the last 35 years remains constant (which appears to be conservative given the accelerated warming of the last decade), then this would result in a 2.5°C increase in mean annual sea surface temperature by 2,100. Making the simplifying assumption that this temperature increase is constant across each day of the entire year, this would result in a 6% annual increase in metabolic requirements, from 1,055 kJ (current) to 1,123 kJ (projected), requiring the consumption of 131 additional bivalves per year. Thus, the ability of green crabs to meet metabolic demands may be compromised in the Gulf of Maine because of the combined impacts of reduced foraging payoff (due to climate change and the invasive Asian shore crab) and increased foraging requirement (imposed by a warming climate). Many ecological systems today are experiencing multiple stressors in marine (Crain, Kroeker, & Halpern, 2008), freshwater (Heugens, Hendriks, Dekker, van Straalen, & Admiraal, 2001) and terrestrial systems (Aber et al., 2001), and these may interact in synergistic ways. Two of the most prevalent stressors today are habitat destruction/deterioration and climate change. Individual green crabs may respond to these multiple stresses by moving subtidally, which requires less energy expenditure for this species than intertidal existence (McDonald et al., 2006). As noted in the Introduction, differences in the number of clutches produced annually in different geographical areas by green crabs (Yamada, 2001) suggest that this species may have some flexibility in the relative use of capital vs. income strategies. If this is the case, and if capital strategies become less energetically favorable due to the cost of stored capital as environmental conditions shift (particularly as the climate warms), it is possible that green crabs may respond adaptively by increasing their relative use of income breeding strategies. However, this possibility remains to be examined.

Finally, it has been suggested that the extra costs of energy storage for capital breeders may enable income breeders that do not share these costs to outcompete capital breeders, especially in poor‐quality habitats where meeting metabolic demands is a challenge (Houston, Stephens, Boyd, Harding, & McNamara, 2007; Stephens et al., 2009). The results here demonstrate that these storage costs may be substantial, suggesting that this theoretical competitive disadvantage for capital breeders may apply to small‐bodied poikilotherms. The Asian shore crab appears to be an income breeder (Griffen, Altman, Bess, Hurley, & Penfield, 2012), and since its invasion in the late 1980s, it has steadily replaced the green crab as the dominant species in rocky intertidal habitats (Lohrer & Whitlatch, 2002). Previous work has posited that this species replacement may be the result of predation by Asian shore crabs on green crab larvae (Lohrer & Whitlatch, 2002) or may stem from decreases in reproductive effort that follow diet shifts in the green crab induced by interactions between these species (Griffen et al., 2011). Results presented here suggest a third mechanism that may be a contributing factor to this species replacement. Specifically, the costs of storing energy to be used in reproduction by green crabs may present an extra cost that makes the green crab an inferior competitor under some conditions.

## Conflict of interest

None declared.
